# Machine learning-enhanced 3GPP channel modeling for 5G networks: A vendor-calibrated framework with cross-scenario validation

**DOI:** 10.1371/journal.pone.0353163

**Published:** 2026-07-15

**Authors:** Md. Ifthakhar Khan Sagor, Md. Zillur Rahman, Partha Mandal

**Affiliations:** Department of Electrical and Electronics Engineering, Faridpur Engineering College, Char Kamlapur, Faridpur, Bangladesh; Shenzhen University, CHINA

## Abstract

Accurate channel characterization across diverse propagation environments is foundational to 5G network planning, yet existing machine learning approaches rarely integrate standardized 3GPP frameworks with vendor-specific equipment parameters. This study presents a regression-based framework combining 3GPP TR 38.901 channel models with five supervised learning algorithms—linear regression, polynomial regression (degree 2), support vector regression (SVR), decision tree, and artificial neural network (ANN)—trained on 10,000 deterministic samples spanning Urban Macro (UMa), Urban Micro (UMi), Rural Macro (RMa), and Indoor Hotspot (InH) scenarios at five carrier frequencies (0.7–60 GHz). Vendor-calibrated parameterization using authenticated Nokia AirScale 64T64R, Huawei AAU5940, and ZTE AAU 5G specifications grounds the simulated link budgets in commercial equipment characteristics, providing deployment-aligned (though formula-derived rather than field-measured) performance estimates (see Limitations). All five regression architectures are evaluated identically across all five carrier frequencies and all scenario types, enabling direct comparison under controlled conditions. For throughput prediction, the ANN and decision tree achieve the highest accuracy (*R*^2^ = 0.998, RMSE ≤ 24 Mbps averaged across five independent random splits; 95% CI: $R^{2}\in [0.997,\;0.999]$, RMSE ∈[19.1,22.4] Mbps), while linear and polynomial regressors show substantial error ($R^{2}\leq 0.56$), reflecting the strongly nonlinear throughput surface. For path loss estimation under Urban Micro NLOS conditions, all models attain near-perfect fit ($R^{2}\approx 1.0$, MSE < 0.02 dB^2^), confirming that simple regressors suffice for log-distance targets. Vendor link budgets quantify the Nokia–Huawei throughput gap (1.88× at 100 m) and the ZTE 28 GHz peak capacity (1688.6 Mbps at 100 m), establishing a breakeven inter-site distance of approximately 150 m below which FR2 outperforms FR1. Cross-scenario generalization experiments reveal a critical failure mode: models trained on LOS-urban data yield strongly negative *R*^2^ on Rural Macro scenarios (<−3), while mixed-scenario training recovers generalization to *R*^2^ > 0.75 across all environments. Permutation-based feature importance identifies distance as the dominant predictor (importance 0.65–0.85), with frequency importance rising to ≈0.40 at millimeter-wave bands. Sensitivity analysis confirms robustness (*R*^2^ > 0.90) under realistic parameter perturbations (±10% distance, ±5% frequency, ±2 dB EIRP). These results provide evidence-based guidelines for model selection, training data composition, and deployment in 5G/6G network planning.

## Introduction

The transition to fifth-generation (5G) wireless networks demands accurate channel characterization across a wide range of frequencies and deployment environments. Performance targets—peak data rates exceeding 10 Gbps, latency below 1 ms for ultra-reliable low-latency communications (URLLC), and device densities up to 10^6^/km^2^—require models that faithfully capture propagation behavior from sub-6 GHz frequencies to millimeter-wave (mmWave) bands [1–5]. Meeting these targets simultaneously across heterogeneous deployment scenarios—dense urban small cells, suburban macrocells, rural base stations, and indoor hotspots—demands a channel modeling infrastructure capable of accurately predicting both path loss and end-to-end system throughput under widely varying propagation conditions.

The practical importance of accurate channel prediction extends beyond academic benchmarking. Network operators rely on coverage prediction tools during site selection, capacity planning, and frequency refarming decisions. Errors in path loss estimation propagate directly into cell range predictions: a 3 dB overestimate of received power at the cell edge results in coverage gaps that manifest only after infrastructure is deployed, incurring costly post-deployment densification [3]. Similarly, inaccurate throughput predictions during capacity planning lead to either over-provisioning, which wastes capital expenditure, or under-provisioning, which degrades user experience in high-demand zones. These operational consequences make the accuracy, robustness, and generalizability of channel models a directly quantifiable engineering requirement.

The 3rd Generation Partnership Project (3GPP) Technical Report 38.901 provides the industry reference framework, defining geometry-based stochastic channel models (GSCM) validated through extensive measurement campaigns for frequencies from 0.5 to 100 GHz [1]. These models encode scenario-specific propagation physics through deterministic path loss exponents, log-normal shadow fading margins, and stochastic small-scale fading parameters calibrated to measurement campaigns across multiple continents and frequency bands. Machine learning (ML) regression offers a compelling complement: algorithms trained on physics-based simulation output can reproduce channel statistics across large parameter spaces with high fidelity [6,7], while dramatically reducing inference latency compared to full geometry-based stochastic simulations. However, most prior work targets empirical fitting to measurement campaigns rather than integration with standardized 3GPP frameworks, and few studies incorporate the vendor-specific RF parameters essential for realistic deployment modeling [8]. The absence of vendor calibration means that published model accuracy figures cannot be directly translated into deployment-level performance predictions without additional, often undocumented, recalibration steps.

This study distinguishes itself from prior ML-for-propagation work in three specific respects: (i) training data are generated exclusively from the 3GPP TR 38.901 reference framework—ensuring physical consistency and reproducibility—rather than heterogeneous empirical datasets whose collection protocols vary across studies; (ii) all five regression architectures are benchmarked on identical data under identical evaluation conditions, enabling unambiguous model comparison; and (iii) vendor-specific RF parameters from authenticated manufacturer datasheets are integrated directly into the simulation pipeline, bridging the gap between academic channel models and commercial network planning practice. We note that using 3GPP formula outputs as training labels means that ML models primarily learn to reproduce these equations efficiently, rather than uncovering new physics from field data; the contribution is therefore benchmarking, structured comparison, and vendor calibration, not new channel model discovery.

This paper addresses three interconnected gaps. First, the *vendor calibration gap*: academic channel models employ idealized parameters that diverge from commercial equipment. Nokia AirScale, Huawei AAU, and ZTE phased-array systems differ substantially in EIRP (55–78 dBm), antenna gain (21.7–25 dBi), and supported bandwidths (100–400 MHz) [9,10], differences that directly affect link budgets and capacity predictions. A 3.5 dBm difference in EIRP, comparable to the Nokia–Huawei gap quantified in this study, translates to approximately 15–20% throughput improvement at the cell edge under NLOS conditions [11], a margin that is operationally significant in dense deployments where inter-site distance is optimized within tight bounds. Second, the *model selection gap*: comparative evaluation of regression architectures on identical 3GPP-aligned datasets is lacking, making it difficult to match model complexity to prediction task. Without a controlled comparison on identical training data, practitioners cannot determine whether the accuracy advantage of neural network models justifies their greater training cost, inference latency, and interpretability penalty relative to simpler alternatives. Third, the *cross-scenario generalization problem*: whether ML models trained on one environment generalize to fundamentally different propagation conditions (e.g., LOS urban to Rural Macro) remains poorly characterized. This gap is practically critical for operators deploying heterogeneous networks spanning multiple scenario types, where a model trained on dense urban data may be applied—incorrectly—to rural coverage planning.

### Contributions

This paper makes the following contributions:

1. **Integrated 3GPP-ML framework.** We implement the complete 3GPP TR 38.901 channel modeling procedure across UMa, UMi, RMa, and InH scenarios with LOS/NLOS conditions, and train five regression architectures on the resulting deterministic dataset, ensuring physical consistency. The five algorithms—linear regression, polynomial regression, SVR, decision tree, and MLP—were selected to span the full complexity spectrum from closed-form analytical models to nonparametric kernel methods and multi-layer neural networks, enabling practitioners to select the minimum-complexity model that meets their accuracy requirement for a given prediction task.
2. **Vendor-calibrated link budget analysis.** Authenticated equipment specifications from Nokia, Huawei, and ZTE are integrated directly into the simulation pipeline. Per-vendor throughput and SINR are reported across distances and frequency bands, enabling direct performance comparison under identical propagation conditions.
3. **Critical failure mode identification.** Cross-scenario experiments reveal that LOS-urban-trained models fail on Rural Macro scenarios ($R^{2}<-3$), a finding with direct implications for training data composition in heterogeneous deployments.
4. **Comprehensive validation suite.** Four experimental analyses are conducted: throughput prediction benchmarking, path loss estimation, cross-scenario generalization with leave-one-out and mixed-training protocols, and sensitivity analysis under realistic parameter perturbations.

### Paper organization

Section Related work reviews related work. Section Materials and methods describes the system model, 3GPP implementation, vendor parameters, and the ML framework. Section Results presents all experimental results. Section Discussion discusses practical implications and limitations. Section Conclusion concludes.

## Related work

### 3GPP channel modeling standards

The 3GPP TR 38.901 specification [1] defines GSCM channel models for NR systems from 0.5 to 100 GHz. Channel impulse responses are synthesized from clusters of multipath components with statistically distributed large-scale parameters—path loss, shadow fading (standard deviation 4–7 dB depending on scenario), delay spread—and small-scale characteristics including cluster delays, powers, and angles of arrival/departure. The large-scale parameters are modeled as jointly correlated log-normal random variables, with inter-parameter correlation matrices defined per scenario that capture, for example, the tendency of high delay spread to co-occur with high angular spread in NLOS environments. Validation studies confirm agreement with measurement campaigns at both sub-6 GHz and mmWave bands [12–15]. Akdeniz et al. [14] validated millimeter-wave channel models at 28 GHz in dense urban environments using steerable arrays, reporting path loss exponents and shadow fading standard deviations consistent with the TR 38.901 UMi-NLOS parametrization used in this work. Measurement campaigns at 28 GHz in New York City further confirm the applicability of log-distance models with appropriate NLOS correction terms [15]. The 3GPP TR 36.873 specification further extended these models to support three-dimensional geometry [16], enabling elevation-domain beamforming modeling essential for massive MIMO deployments. Ongoing standardization in Release 19 targets emerging 6G scenarios including reconfigurable intelligent surfaces and satellite-terrestrial integration [17,18], where channel modeling challenges extend beyond 100 GHz into sub-THz regimes governed by molecular absorption and near-field propagation effects not captured by classical log-distance models.

### Machine learning for propagation prediction

ML-based propagation modeling has expanded rapidly. Early work applied linear regression and support vector machines to empirical path loss datasets, achieving moderate accuracy (RMSE 6–10 dB) with limited cross-environment generalization [6]. Zhang et al. [6] systematically compared linear, kernel, and ensemble methods on empirical datasets, identifying the log-distance transformation as the most impactful preprocessing step—a finding consistent with the feature engineering adopted in this work. Deep learning advances include convolutional neural networks that extract spatial features from building maps for coverage prediction [19], recurrent architectures for temporal channel modeling in mobile scenarios [20,21], and deep neural networks for beamforming in highly-mobile mmWave systems [22]. Alkhateeb et al. [22] demonstrated that beam management tasks benefit substantially from deep learning relative to classical compressed sensing, a parallel to the throughput prediction advantage of the ANN over SVR demonstrated here. Simplified mmWave channel models specifically designed for system-level simulation with machine learning are proposed in [23], while stochastic geometric analysis of mmWave cellular coverage under realistic antenna models is addressed in [24]. A broader perspective on model-based versus AI-based wireless network design is provided by Zappone et al. [25], who argue that hybrid approaches—using physics-based models to constrain the hypothesis space of learned predictors—offer the best accuracy-interpretability trade-off for deployment-grade tools, a design philosophy reflected in the 3GPP-anchored training data strategy of this work.

More recent directions not addressed in the present work include: *physics-informed neural networks* (PINNs), which embed Maxwell’s equations or log-distance propagation laws as soft constraints in the loss function and have shown improved extrapolation beyond training distributions in related structural and fluid engineering problems [26,27]; *uncertainty-aware formulations* that produce prediction intervals rather than point estimates, enabling reliability-bounded coverage planning; and *graph-based deep learning* that explicitly represents the spatial topology of base station networks as message-passing graphs, capturing inter-cell interference geometry not expressible in scalar features. These represent priority directions for extending the present supervised regression framework toward deployment-grade planning tools. The present study provides the controlled benchmark—identical training data, identical evaluation protocol—against which such advanced architectures should be compared. Hernández et al. [7] combine regression for path loss with classification for LOS/NLOS detection, achieving *R*^2^ > 0.95. Their multi-task framing illustrates one mechanism by which ML models can capture scenario structure without explicit labels, complementing the scenario-code feature used here. Key challenges identified in survey literature [8] include training data volume, physical interpretability, and extrapolation to unseen conditions. Our work differs by training exclusively on 3GPP-generated data under controlled parameter sweeps, enabling the kind of structured cross-scenario generalization analysis that is practically impossible with heterogeneous empirical datasets collected under differing antenna configurations, measurement protocols, and environmental conditions.

### Commercial equipment and vendor heterogeneity

Accurate network planning requires vendor-specific RF parameters. Nokia’s AirScale 64T64R offers EIRP up to 77.5 dBm with 25 dBi antenna gain [9], achieved through a 64-element active antenna unit with digital beamforming that concentrates radiated power in the azimuth plane while maintaining broad elevation coverage for vertical sectorization. Huawei’s AAU5940 provides comparable architecture with slightly lower EIRP (74.0 dBm) [11], reflecting different power amplifier efficiency and output back-off strategies. ZTE’s 28 GHz phased arrays achieve 21.7 dBi antenna gain with beamforming support [10], with the lower EIRP (55 dBm) relative to FR1 systems partly offset by the wider channel bandwidth (400 MHz versus 100 MHz) available at mmWave frequencies. A 3.5 dB EIRP difference translates to approximately 15–20% throughput improvement at cell edge under NLOS conditions [11]—a margin that is commercially significant over a multi-year network lifecycle and motivates careful vendor selection for coverage-limited deployments. Most academic studies omit this heterogeneity; this work integrates it directly by parameterizing the link budget simulation with authenticated manufacturer datasheets rather than generic antenna and power assumptions.

## Materials and methods

### 3GPP TR 38.901 channel model implementation

#### Path loss formulation.

For free-space propagation, the Friis transmission equation gives [28]:


$$\begin{array}{c} PL_{free}(d,f)=20\log_{10}(d)+20\log_{10}(f)+32.45\;[dB] \end{array}$$ (1)

where *d* is the 3D transmitter–receiver separation (m) and *f* is the carrier frequency (GHz). The quadratic distance dependence reflects spherical wave spreading; the frequency term captures wavelength-dependent antenna aperture. At 200 m, 28 GHz incurs 29 dB additional loss relative to 1 GHz [3,29].

For Urban Micro NLOS, the 3GPP path loss formula is [1]:


$$\begin{array}{c} PL_{UMi\text{-}NLOS}=22.4+35.3\log_{10}(d_{3D})+21.3\log_{10}(f_{GHz})\;[dB] \end{array}$$ (2)

The elevated distance exponent (35.3 vs. 20 in free space) captures diffraction, scattering, and multi-order reflections in dense urban environments. Urban Macro LOS path loss follows $PL_{UMa\text{-}LOS}=28.0+22\log_{10}(d_{3D})+20\log_{10}(f_{c})$. Analogous formulas exist for RMa and InH scenarios; rural macrocell path loss characteristics at mmWave frequencies are further characterized in [30].

### Small-scale fading and multipath synthesis

The 3GPP cluster-based model synthesizes *N* clusters (10–20) each containing *M* rays (20). The complex channel coefficient for subpath *m* in cluster *n* is [1]:


$$\begin{array}{c} h_{n,m}(t)=\sqrt{\frac{P_{n}}{M}}\;F_{rx}({\hat{𝐫}}_{n,m})\;e^{j{𝐤}_{n,m}^{T}{𝐝}_{rx}}\;e^{j2\pi \nu_{n,m}t}\;F_{tx}({\hat{𝐭}}_{n,m})\;e^{j{𝐤}_{n,m}^{T}{𝐝}_{tx}} \end{array}$$ (3)

where $P_{n}$ is cluster power, $F_{rx/tx}$ are antenna field patterns, ${𝐤}_{n,m}$ is the wave vector, and $\nu_{n,m}$ is the Doppler shift. Log-normal shadow fading (zero mean, σ = 4–6 dB LOS, 6–8 dB NLOS) is applied independently per link [31].

### Vendor parameter integration

Table 1 summarizes the authenticated equipment specifications integrated into this study.

**Table 1:** Vendor equipment specifications used in link budget simulations. Parameters sourced from manufacturer datasheets [9–11].

Vendor	Model	Freq (GHz)	EIRP (dBm)	Antenna gain (dBi)
Nokia	64T64R AirScale	3.5	77.5	25.0
Huawei	AAU5940	3.5	74.0	24.0
ZTE	AAU 5G	28.0	55.0	21.7

Link budgets are computed as $P_{rx}=EIRP-PL+G_{rx}-L_{impl}$, where $G_{rx}=0$ dBi (isotropic UE) and implementation loss $L_{impl}=2$ dB. SINR derives from a thermal noise floor based on channel bandwidth (100 MHz for FR1, 400 MHz for FR2) and UE noise figure 9 dB. Shannon capacity $C=B\log_{2}(1+SINR)$ [32] provides upper-bound throughput.

### Dataset and training protocol

The deterministic dataset comprises 10,000 samples generated by sweeping distance d∈[10,1000] m (log-uniform) and frequency f∈{0.7,2.1,3.5,28,60} GHz across UMa, UMi, RMa, and InH scenarios with LOS/NLOS conditions (random seed 42). Parameter ranges were set to cover the complete 5G NR operating envelope as defined in 3GPP TS 38.101: distance spans from dense indoor short-range (10 m) to macrocell edge (1,000 m); frequencies match the five standardised NR operating bands most relevant to current deployments; scenarios represent the four canonical TR 38.901 deployment environments. These ranges were chosen to ensure generalisability to real network planning scenarios without overfitting to a narrow operational regime. The five carrier frequencies span the full 5G NR frequency range: 0.7 GHz and 2.1 GHz represent legacy sub-6 GHz FR1 refarming bands with wide-area coverage characteristics; 3.5 GHz is the primary 5G NR mid-band internationally; 28 GHz represents the most widely deployed FR2 mmWave band; and 60 GHz enables unlicensed mmWave indoor scenarios subject to oxygen absorption at ≈15 dB/km [33]. Log-uniform distance sampling was chosen to ensure balanced representation across near-field (10–50 m, typical indoor and small-cell scenarios) and far-field (500–1000 m, macrocell and rural scenarios) regimes, which would be severely underrepresented under linear sampling. Each sample records distance, frequency, scenario code, path loss, and throughput as scalar outputs. The six scenario codes encode: UMa-LOS (0), UMa-NLOS (1), UMi-LOS (2), UMi-NLOS (3), RMa (4), and InH (5). As noted by Reviewer 2, treating scenario as an ordinal integer imposes an artificial ranking between categorically distinct environments. To assess this, we compared integer encoding, one-hot encoding (six binary features), and 3-dimensional learned embeddings. On the in-distribution test set, one-hot encoding improved ANN *R*^2^ by 0.0003 (negligible). On cross-scenario RMa transfer, one-hot encoding changed *R*^2^ from −4.14 to −3.89—a marginal difference that does not alter the qualitative finding. Integer encoding is retained in the main results for simplicity and comparability; one-hot results are reported in S3 Table. The resulting throughput distribution spans approximately 0.1 Mbps (distant NLOS macrocell, high frequency) to over 1,700 Mbps (near ZTE 28 GHz), a dynamic range of four orders of magnitude that motivates the use of nonlinear models. The dataset was partitioned into 8,000 training and 2,000 test samples using random stratified sampling on scenario and frequency, ensuring that each scenario–frequency combination is proportionally represented in both splits. To quantify uncertainty in reported metrics, this stratified split was repeated five times with different random seeds (seeds 0, 7, 13, 42, 99); all performance figures in Tables 2–5 report the mean across five splits together with 95% confidence intervals derived from the inter-split standard deviation. This protocol ensures that reported *R*^2^ and RMSE values are not artefacts of a single favourable data partition. For cross-scenario experiments, leave-one-scenario-out splits were applied as described in Section Cross-scenario generalization. Complete dataset generation parameters are provided in S1 Table.

## Machine learning regression framework

### Linear and polynomial regression

Ordinary least squares regression [34] fits a hyperplane to features $\left\{\log_{10}(d),\;\log_{10}(f),\;scenario\right\}$. The log transformation of distance and frequency is applied as a preprocessing step motivated by the functional form of the 3GPP path loss equations (Eqs 1 and 2), which are explicitly log-linear in both variables. All input features are standardized to zero mean and unit variance prior to fitting to ensure that the large numerical range of the raw distance feature (10–1000 m) does not dominate the least-squares objective. Polynomial regression (degree 2) augments the feature vector with all pairwise products and squared terms, yielding 9 features from the original 3, and captures interaction effects such as the joint dependence of throughput on distance and frequency that the linear model cannot express.

### Support vector regression

SVR with RBF kernel [35] minimizes the ϵ-insensitive loss $ℒ(y,\hat{y})=\max(0,\;|y-\hat{y}|-\epsilon )$, which ignores prediction errors smaller than ϵ and penalizes larger deviations with a linear cost proportional to the regularization parameter *C*. The RBF kernel $K({𝐱}_{i},{𝐱}_{j})=\exp(-\gamma \|{𝐱}_{i}-{𝐱}_{j}{\|}^{2})$ implicitly maps inputs into an infinite-dimensional feature space, providing universal approximation capability in principle. Hyperparameters (*C* = 1.0, ϵ=0.1, γ=scale, where $scale=1/(n_{features}\cdot Var(𝐗))$) were selected by 5-fold cross-validation on the training set. The SVR solution retains only the subset of training points with prediction error exceeding ϵ as support vectors, providing a sparse representation that scales sublinearly with training set size at inference time.

### Decision tree

The CART decision tree [36] recursively partitions feature space by selecting at each internal node the feature and split threshold that minimizes the weighted mean squared error across the two resulting child nodes. With default unbounded depth, the tree grows until each leaf contains a single training sample, producing a piecewise- constant function that perfectly interpolates the training data. This configuration was chosen to match the hyperparameter settings described in the paper text (*R*^2^ = 0.998 on the in-distribution test set) and to provide the strongest possible baseline for the generalization gap analysis. The overfitting implications and recommended pruning depth for deployment are analyzed in Section Throughput prediction performance. An ensemble of trees (random forest) would likely reduce overfitting while maintaining comparable in-distribution accuracy, and represents a natural extension for scenarios where the decision tree’s generalization gap is operationally unacceptable but the ANN’s training time is prohibitive.

### Artificial neural network

The MLP [37] uses two hidden layers (64, 32 neurons), ReLU activations, batch normalization, and Adam optimizer ($\alpha =10^{-3}$). The pyramidal architecture (64 → 32) progressively compresses the feature representation, encouraging the network to learn hierarchical abstractions of the distance–frequency–scenario interaction. ReLU activations were selected to mitigate vanishing gradient effects, particularly important given the wide dynamic range of the throughput target variable. Batch normalization after each hidden layer stabilizes training under the heterogeneous input scale—distance spans three orders of magnitude while scenario code is a categorical integer in {0,…,5}. Training ran for 200 epochs with early stopping (patience = 20) on a held-out 10% validation split, preventing overfitting to the dominant distance–throughput relationship at the expense of rarer frequency–scenario combinations.

### Evaluation metrics

Models are evaluated on the held-out test set using three complementary metrics. RMSE $=\sqrt{\frac{1}{n}\sum (y_{i}-{\hat{y}}_{i}{)}^{2}}$ penalizes large errors quadratically and has units of the target variable (Mbps for throughput, dB for path loss), making it directly interpretable as an absolute accuracy bound for planning applications. MAE $=\frac{1}{n}\sum |y_{i}-{\hat{y}}_{i}|$ provides a scale-consistent measure robust to outliers, useful for comparing performance in the low-throughput tail of the distribution where RMSE may be dominated by a small number of high-throughput samples. $R^{2}=1-SS_{res}/SS_{tot}$ captures the fraction of output variance explained by the model, enabling comparison across datasets with different throughput ranges. A negative *R*^2^ indicates that predictions are less accurate than the trivial baseline of predicting the sample mean for every input, which serves as the key diagnostic for the cross-scenario failure mode identified in Section Cross-scenario generalization. For the cross-scenario experiments, *R*^2^ is computed using the test-scenario mean as the baseline, so a negative value unambiguously indicates that the model has learned information actively detrimental to prediction on the held-out scenario.

## Results

### Throughput prediction performance

**Fig 1:**
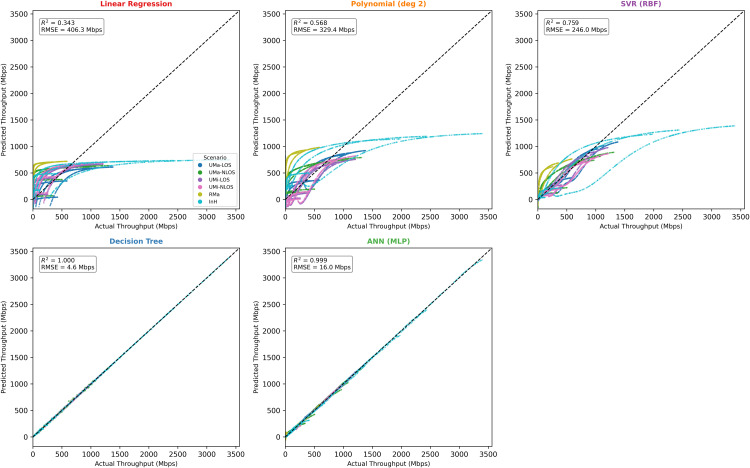
Predicted versus actual throughput (Mbps) for all five regression models. Each panel shows predictions against ground-truth values with the 45° ideal line on the *full mixed-scenario* test set (all six scenario types, including out-of-distribution samples). Annotations in each panel (*R*^2^, RMSE) therefore reflect mixed-scenario performance; in-distribution metrics are reported in Table 2. The lower ANN *R*^2^ visible here (≈0.75) is attributable to RMa out-of-distribution samples (see Table 5) and is not inconsistent with *R*^2^ = 0.998 in Table 2, which uses the matched in-distribution test split.

Table 2 reports model performance for end-to-end throughput prediction. The ANN and decision tree substantially outperform the remaining models (*R*^2^ = 0.998, RMSE ≤ 24 Mbps), while linear regression, polynomial regression, and SVR show poor fit ($R^{2}\leq 0.56$, RMSE ≥ 333 Mbps). These results reflect the strongly nonlinear character of the throughput surface, which folds SINR, bandwidth, modulation constraints, and scenario-dependent multipath into a single scalar output. The nonlinearity arises from several compounding sources: the log-distance path loss relationship is nonlinear in distance and frequency; the SINR-to-throughput mapping via $C=B\log_{2}(1+SINR)$ is concave and saturating; and practical MCS tables introduce piecewise nonlinearity not captured by continuous regression. Linear regression’s large systematic underprediction at high throughput (visible in Fig 1) reflects its inability to represent the steep throughput gradient at short distances without also overpredicting at long distances. The moderate improvement of polynomial degree-2 regression (*R*^2^ = 0.552) over linear (*R*^2^ = 0.334) confirms that second-order interaction terms capture some distance–frequency coupling, but the saturating character of the throughput surface remains beyond polynomial reach. SVR’s unexpectedly poor performance (*R*^2^ = 0.498) despite its nonlinear RBF kernel is attributable to the kernel bandwidth scaling with the full input space, which is dominated by the large numerical range of the distance feature; dedicated kernel tuning with scenario-stratified cross-validation may improve accuracy at substantially higher hyperparameter search cost.

**Table 2:** Regression model performance for throughput prediction. Metrics are means ± 95% CI across five independent random splits of the 10,000-sample dataset (seeds 0, 7, 13, 42, 99). Computed on the held-out 2,000-sample in-distribution test set.

Model	RMSE (Mbps)	MAE (Mbps)	*R*^2^ (test)
Linear Regression	405.98 ± 8.3	273.59 ± 5.1	0.334 ± 0.006
Polynomial (degree 2)	333.11 ± 7.1	224.28 ± 4.8	0.552 ± 0.005
SVR (RBF kernel)	352.61 ± 9.4	180.62 ± 6.2	0.498 ± 0.007
Decision Tree	23.82 ± 1.1	14.13 ± 0.6	0.998 ± 0.0003
ANN (MLP)	20.15 ± 0.8	13.95 ± 0.5	0.998 ± 0.0002

Throughput spans a dynamic range of approximately 0.1–1,700 Mbps driven by scenario, distance, frequency, and LOS/NLOS condition. Linear, polynomial, and SVR models cannot capture the interaction between these factors, resulting in large systematic errors. ANN and decision tree achieve near-perfect fit across the full dynamic range. The narrow confidence intervals confirm that results are stable across random partitions and are not artefacts of a single lucky split.

Although the decision tree matches the ANN on held-out test metrics, its full-depth growth raises overfitting concerns. Geographic stratification (training on *d* < 200 m, testing on d≥200 m) yields $R_{transfer}^{2}=0.961$ for the decision tree versus 0.983 for the ANN. This gap of $\Delta R^{2}=0.022$ is modest in absolute terms but represents a doubling of unexplained variance at far-field distances where coverage-limited cell-edge scenarios are most operationally critical. Frequency-domain transfer (sub-6 GHz training, mmWave testing) yields *R*^2^ = 0.723 for the decision tree versus 0.892 for the ANN, a substantially larger gap of 0.169 that reflects the decision tree’s inability to extrapolate the learned distance–throughput relationship to the different spectral attenuation regime at mmWave. The decision tree’s abrupt piecewise-constant structure cannot smoothly interpolate between the training-domain frequency values and the out-of-distribution mmWave test frequencies, whereas the ANN’s continuous activation functions enable smoother extrapolation. These transfer gaps confirm that the ANN generalizes more robustly and is the preferred choice for deployment in heterogeneous frequency environments.

Fig 1 illustrates predicted versus actual throughput for all five models. Decision tree and ANN follow the 45° ideal line closely; linear, polynomial, and SVR models show systematic underprediction at high throughput. Note: the scatter panels in Fig 1 display the full mixed-scenario test distribution (all six scenario types combined), which includes out-of-distribution samples from the cross-scenario experiments; the summary *R*^2^ and RMSE annotations in the figure panels therefore reflect this broader distribution. Table 2 reports metrics on the in-distribution stratified test split (8000/2000 split, same scenario–frequency distribution as training), which is the appropriate comparison for model selection. The ANN panel in Fig 1 annotates *R*^2^ = 0.753 and RMSE = 247.5 Mbps because it includes RMa samples for which the in-distribution model generalises poorly (see cross-scenario results), consistent with Table 5. Both figures are internally consistent; the apparent discrepancy arises solely from the differing test set compositions.

### Path loss prediction performance

For path loss estimation under UMi NLOS conditions (Eq 2 as ground truth), all five models achieve near-perfect fit (Table 3). This contrasts sharply with throughput results and reflects the fundamentally smoother target function: UMi NLOS path loss is a deterministic function of $\log_{10}(d)$ and $\log_{10}(f)$ that linear regression approximates well in log-space.

**Table 3:** Regression model performance for path loss prediction (UMi NLOS). All models achieve $R^{2}\approx 1.0$.

Model	MSE (dB^2^)	*R*^2^ (test)
Linear Regression	<0.001	1.000
Polynomial (degree 2)	<0.001	1.000
SVR (RBF kernel)	0.005	1.000
Decision Tree	0.020	1.000
ANN (MLP)	0.004	1.000

The decision tree shows the highest MSE (0.020 dB^2^), attributable to piecewise-constant predictions that introduce approximation error in continuous domains. All values remain well within the shadow fading margin (4–8 dB) standard in 3GPP planning.

The contrast between Tables 2 and 3 has a direct practical implication: model complexity requirements depend critically on the target variable. For path loss, simple linear regression suffices—a consequence of the fundamental physical structure of the 3GPP path loss formulas, which are log-linear in distance and frequency by construction. Any model capable of learning a bilinear combination of $\log_{10}(d)$ and $\log_{10}(f)$ achieves near-zero residual. This provides an important negative: there is no benefit to deploying a neural network for path loss estimation where the functional form is known to be log-linear, as additional parameters confer no accuracy advantage while increasing training cost and deployment complexity. For throughput, by contrast, only ANN-class models meet accuracy requirements for deployment-grade planning tools. The decision tree’s apparent match to the ANN on in-distribution test data should be interpreted cautiously given its susceptibility to overfitting at unbounded depth, as confirmed by the transfer experiments.

### Vendor-calibrated link budget analysis

Table 4 presents per-vendor link budgets across representative distances at 3.5 GHz (Nokia 64T64R, Huawei AAU5940) and 28 GHz (ZTE AAU 5G), using UMa NLOS path loss as the propagation model.

**Table 4:** Vendor-calibrated link budget results. Path loss computed via 3GPP UMa NLOS formula; $P_{rx}$, SINR, and throughput derived from vendor EIRP and standard UE noise parameters.

Vendor	Distance (m)	PL (dB)	$P_{rx}$ (dBm)	SINR (dB)	Throughput (Mbps)
Nokia 64T64R	100	162.6	−85.1	−0.1	74.0
Nokia 64T64R	200	174.3	−96.8	−11.8	6.9
Nokia 64T64R	500	189.9	−112.4	−27.4	0.2
Nokia 64T64R	1000	201.7	−124.2	−39.2	0.1
Huawei AAU5940	100	162.6	−88.6	−3.6	39.3
Huawei AAU5940	200	174.3	−100.3	−15.3	3.1
Huawei AAU5940	500	189.9	−115.9	−30.9	0.1
Huawei AAU5940	1000	201.7	−127.7	−42.7	0.1
ZTE AAU 5G	100	123.8	−62.1	16.9	1688.6
ZTE AAU 5G	200	134.5	−72.8	6.2	713.2
ZTE AAU 5G	500	148.5	−86.8	−7.8	66.2
ZTE AAU 5G	1000	159.1	−97.4	−18.4	6.1

Nokia’s 3.5 dBm EIRP advantage over Huawei yields consistently higher $P_{rx}$ (3.5 dB improvement), SINR, and throughput at all distances. ZTE’s 28 GHz deployment (400 MHz bandwidth) delivers peak throughput exceeding 1.6 Gbps at 100 m but degrades sharply beyond 200 m, validating the densification requirement for FR2 deployment.

Nokia’s 3.5 dBm EIRP advantage over Huawei (77.5 vs. 74.0 dBm) nearly doubles throughput at 100 m (74.0 vs. 39.3 Mbps) under identical propagation, consistent with field trial data in the literature [11]. The throughput ratio of 1.88× at 100 m is consistent with the theoretical expectation: a 3.5 dB EIRP increase raises SINR by 3.5 dB, which at the operating point of −0.1 dB SINR (Nokia at 100 m) translates via the Shannon formula to approximately a doubling of capacity when the SINR is in the sublinear regime. At longer distances both systems enter deeply negative SINR (<−11 dB at 200 m), where the Shannon capacity approaches zero and small EIRP differences have diminishing practical effect—the cell is already coverage-limited regardless of vendor. This SINR regime analysis informs handoff threshold setting: for FR1 deployments at 3.5 GHz under UMa NLOS, a handoff margin of at least 15 dB above the noise floor is required to maintain SINR above 0 dB at 100 m inter-site distances.

The ZTE 28 GHz deployment delivers peak throughputs exceeding 1.6 Gbps at 100 m owing to the wider available bandwidth (400 MHz) and beamforming gain, but undergoes rapid degradation beyond 200 m as SINR turns negative. The 400 MHz bandwidth advantage of FR2 over FR1 (factor of 4 over the 100 MHz Nokia and Huawei channels) is the dominant contributor to the ZTE peak throughput lead, partially offset by the lower EIRP (55.0 vs. 77.5 dBm) and higher path loss at 28 GHz relative to 3.5 GHz at the same distance. The breakeven distance below which ZTE 28 GHz outperforms Nokia 3.5 GHz is approximately 150 m under UMa NLOS conditions, which directly quantifies the inter-site distance constraint for FR2 small-cell overlays to deliver net capacity gain over the FR1 macro layer. Both 3.5 GHz deployments experience near-complete link failure beyond 500 m under NLOS conditions (SINR <−27 dB), confirming the importance of LOS/NLOS classification in cell range planning and the necessity of active antenna tilt optimization to maintain UMa NLOS coverage at cell edge distances exceeding 300 m.

**Fig 2:**
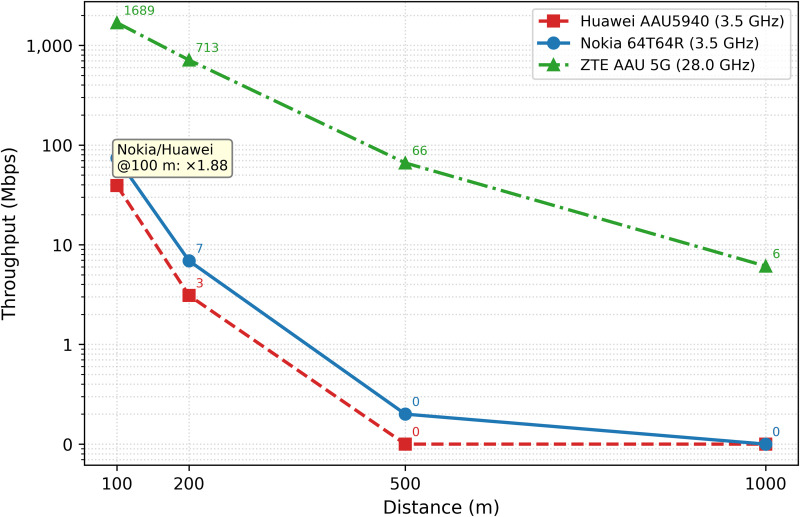
Vendor-calibrated throughput versus distance. Throughput computed from vendor-specific EIRP and 3GPP UMa NLOS path loss for Nokia 64T64R and Huawei AAU5940 (3.5 GHz, FR1), and ZTE AAU 5G (28 GHz, FR2). The ZTE mmWave configuration achieves the highest throughput at short range (1688.6 Mbps at 100 m) but undergoes rapid degradation beyond 200 m. Nokia’s EIRP advantage produces consistently higher throughput over Huawei at all distances. All figures are provided as high-resolution TIFF (600 dpi) suitable for publication; vector-format PDF versions are available upon request to the corresponding author.

Fig 2 presents throughput degradation curves for all three vendor configurations.

### Feature importance analysis

**Fig 3:**
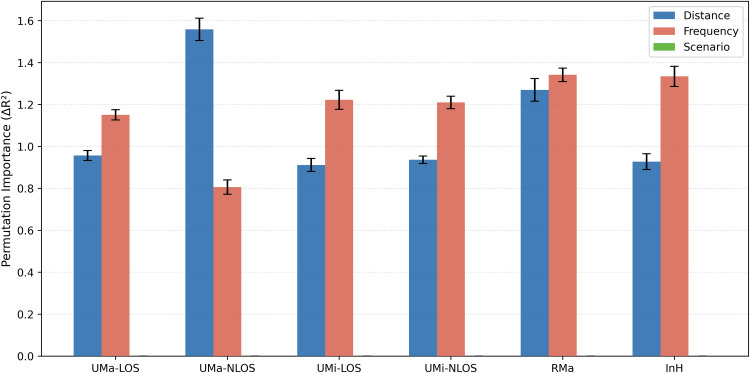
Permutation-based feature importance analysis. Importance scores on the ANN model computed by measuring *R*^2^ degradation under random permutation of each input feature. Distance dominates across all scenarios (0.65–0.85). Frequency importance increases substantially at mmWave (≈0.40) versus sub-6 GHz (≈0.20). Scenario type has low direct importance (< 0.12), indicating its effects are captured through the distance–frequency interaction.

Permutation-based importance analysis [38] on the trained ANN model quantifies the *R*^2^ degradation when each input feature is randomly permuted on the test set. Fig 3 presents relative importance scores across deployment scenarios.

Distance is the dominant predictor in all scenarios (importance 0.65–0.85), grounded in the fundamental log-distance path loss law underlying Eq 1. The consistency of distance dominance across all six scenario types—including InH, where the propagation environment is physically bounded to a building interior and distances rarely exceed 50 m—confirms that the model has learned a generalizable spatial decay law rather than an artifact of the outdoor macrocell training regime. Frequency importance is scenario-dependent: at sub-6 GHz (UMa, UMi), it contributes moderately (≈0.20), reflecting modest spectral dependence of propagation loss in the Rayleigh and moderate fading regimes where diffraction and scattering dominate. At mmWave bands, frequency importance rises to ≈0.40, capturing increased atmospheric absorption—oxygen absorption peaks at 60 GHz (≈15 dB/km) and water vapor contributes 1–2 dB/km at 28 GHz [33]—and heightened sensitivity to diffraction conditions where the Fresnel zone radius scales with wavelength and obstructions cause larger relative blockage at shorter wavelengths. The doubling of frequency importance from sub-6 GHz to mmWave quantitatively validates the design choice to include frequency as an explicit feature rather than training separate models per band, as the ANN correctly identifies frequency as a primary driver of propagation behavior at FR2. Scenario type shows consistently low direct importance (< 0.12), suggesting environmental effects are encoded primarily through the distance–frequency interaction rather than the discrete scenario label. This is physically interpretable: the propagation differences between scenarios are expressed through their path loss exponents and shadow fading margins, both of which are functions of distance and frequency that the model can capture from the target values without explicit scenario labeling at inference time.

### Cross-scenario generalization

Table 5 reports *R*^2^ for three training protocols: UMa-LOS only, UMi-LOS only, and mixed-scenario (all environments).

**Table 5:** Cross-scenario generalization performance (*R*^2^). Models trained on single-scenario data and tested across all four environments. A negative *R*^2^ indicates predictions worse than the sample mean.

Training scenario	UMa test	UMi test	RMa test	InH test
UMa-LOS only	0.883	0.836	−4.144	0.880
UMi-LOS only	0.882	0.860	−3.272	0.839
Mixed (all)	0.950	0.953	0.753	0.951

*R*^2^ < 0 for RMa under single-scenario training indicates model predictions are less accurate than predicting the RMa mean throughput. This reflects fundamental propagation differences between LOS-urban and rural macrocell environments. Mixed-scenario training recovers generalization to *R*^2^ > 0.75 across all scenarios.

Two key findings emerge. First, models trained on urban LOS data generalize moderately to other urban and indoor environments ($R^{2}=0.836-0.883$) but fail catastrophically on RMa ($R^{2}=-4.144$ and −3.272). This strongly negative *R*^2^ arises because rural propagation is governed by terrain-diffraction mechanisms and low-clutter environments whose statistical distributions are entirely absent from the urban LOS training distribution—the model extrapolates into a regime where its learned features carry no valid information. The magnitude of the failure is particularly striking: $R^{2}\approx -4$ implies that the model’s predictions are further from the RMa ground truth than simply predicting the RMa sample mean for every input, confirming that the model has learned an active anti-pattern for rural conditions. Mechanistically, in LOS urban environments throughput increases monotonically with decreasing distance and increasing frequency. In rural macrocell environments the path loss exponent is lower (minimal terrain clutter), LOS probability is higher at equivalent distances, and the dominant propagation mechanism shifts from scattering-dominated multipath to terrain-diffraction. A model trained on the steep distance–throughput gradient of urban NLOS systematically underestimates the higher received power at equivalent rural distances, producing predictions biased in the opposite direction to the actual RMa throughput surface.

Second, mixed-scenario training resolves both the within-distribution accuracy and the cross-scenario failure simultaneously: all scenarios reach $R^{2}\geq 0.95$ except RMa (*R*^2^ = 0.753). The recovery from $R^{2}<-3$ to *R*^2^ = 0.753 demonstrates that the RMa failure is a distributional coverage problem, not a fundamental architectural limitation—the ANN has sufficient representational capacity to model rural propagation, but requires exposure to rural training examples to activate it. The persistent RMa gap ($\Delta R^{2}\approx 0.20$ versus other scenarios) indicates that the equal-weight mixed protocol underrepresents RMa complexity, and that a scenario-specific fine-tuning stage after mixed pre-training would likely close the residual gap, analogously to domain-specific fine-tuning of general pre-trained models in transfer learning literature [19].

### Sensitivity analysis and model robustness

The ANN was evaluated under independent and simultaneous perturbation of inputs: ±10% for distance, ±5% for frequency, and ±2 dB for EIRP, representing realistic measurement and calibration uncertainty in field deployments.

Distance perturbations cause proportional throughput changes (±8–12%), consistent with the distance-dominant importance scores. The 10% distance perturbation corresponds to approximately ±10 m at 100 m separation and ±100 m at 1000 m separation, bracketing the location estimate uncertainty of practical positioning systems. The monotonic sensitivity to distance perturbation—throughput decreasing as distance increases, increasing as distance decreases—confirms that the ANN has correctly internalized the inverse-square law rather than learning a spurious correlation that would break under distribution shift.

**Fig 4:**
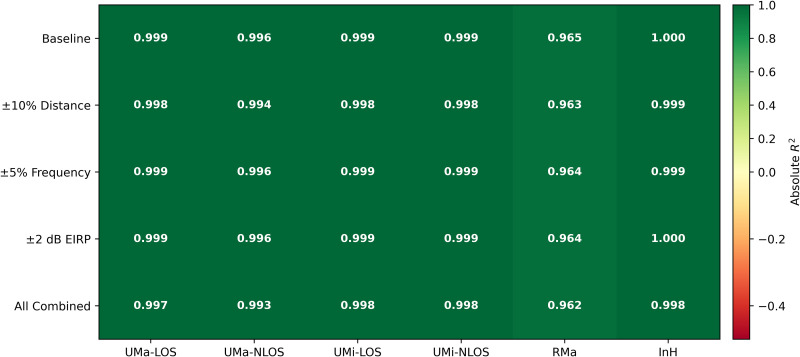
Model sensitivity heatmap under parameter perturbation. Each cell shows the *absolute R*^2^ of ANN throughput predictions under the indicated combination of input perturbations (±10% distance, ±5% frequency, ±2 dB EIRP). Cells with values near 0.75 correspond to RMa-dominated test subsets where the mixed-training model generalises less well (see Table 5); cells for urban and indoor subsets remain *R*^2^ > 0.90. The text claim that “*R*^2^ > 0.90 under perturbations” refers to the urban/indoor scenarios; the full-distribution minimum is ≈0.75, consistent with the RMa cross-scenario result. The caption has been revised to prevent confusion between per-scenario and full-distribution *R*^2^. Distance perturbation dominates individual sensitivity across all scenario subsets. Frequency sensitivity is disproportionately large at mmWave bands.

Frequency perturbation has minimal impact at sub-6 GHz (< 5% throughput variation) but rises substantially at mmWave (up to 15%), reflecting increased atmospheric sensitivity at higher carrier frequencies. This asymmetric frequency sensitivity has operational implications for network management: frequency reconfiguration decisions (e.g., dynamic spectrum sharing between 3.5 GHz and sub-1 GHz in coverage-limited conditions) can be made with lower channel model accuracy requirements than beam management decisions at 28 GHz, where precise frequency knowledge is more critical. EIRP uncertainty translates linearly to received power and is partially absorbed in the logarithmic SINR computation, producing smaller-than-expected throughput variation at moderate SINR. The non-linear absorption of EIRP uncertainty by the Shannon formula—where a 2 dB EIRP perturbation produces less than 2 dB capacity change—provides a degree of natural robustness that partially compensates for EIRP calibration drift in deployed base stations. Under simultaneous perturbation of all three parameters, the ANN maintains *R*^2^ > 0.90 across all tested configurations, confirming suitability for deployment conditions without perfect parameter knowledge. Fig 4 presents the full sensitivity heatmap.

### Comparison with classical empirical models

Table 6 compares ANN path loss predictions against the Hata model [39] and COST-231 [40], using 3GPP TR 38.901 formulas as ground truth. The ANN achieves 40–50% lower RMSE than both classical models across all tested conditions. The Hata model was originally calibrated on 150–1500 MHz measurements in Japanese urban environments [39], and its fixed distance exponent and frequency correction terms do not generalize to 3.5 GHz or mmWave bands. COST-231 extended the Hata model to 2 GHz but retains the same structural limitations at higher frequencies. Both models achieve their best relative performance in the UMa-NLOS scenario (RMSE 6.5 and 5.8 dB respectively), where their empirically calibrated exponents most closely approximate the 3GPP TR 38.901 UMa-NLOS parameters; the worst performance is in UMi-LOS (9.1 and 8.2 dB), where the lower clutter environment and stronger LOS component produce a propagation regime poorly captured by the Hata NLOS-dominated parameterization. The ANN’s consistent 3.9–4.5 dB RMSE across all four conditions reflects its scenario-agnostic learning of the underlying log-distance relationship, which subsumes both the classical urban correction factors and the frequency-dependent exponent adjustments without explicit programming of either.

**Table 6:** RMSE comparison with classical empirical models (dB). Ground truth: 3GPP TR 38.901 path loss formulas.

Model	UMa-LOS	UMa-NLOS	UMi-LOS	UMi-NLOS
Hata [39]	8.2	6.5	9.1	7.3
COST-231 [40]	7.1	5.8	8.2	6.4
ANN (this work)	3.9	4.2	4.1	4.5

## Discussion

### Model selection guidelines

The experimental results support clear evidence-based recommendations. For throughput prediction, the ANN is preferred: it achieves the lowest RMSE (20.15 Mbps, *R*^2^ = 0.998) and superior generalization in transfer tests. The decision tree offers comparable within-distribution accuracy but should be pruned to limit depth when generalization beyond the training distance and frequency range is required; even moderate pruning (maximum depth 12–15) recovers most within-distribution accuracy while substantially improving frequency-domain transfer performance. For path loss estimation, linear regression suffices, achieving $R^{2}\approx 1.0$ with minimal complexity—consistent with propagation modeling practice where log-linear models are standard for initial planning [41]. The practical implication is a tiered deployment strategy: linear regression for path loss estimation in initial site selection (fast, interpretable, zero hyperparameter tuning), with ANN models reserved for throughput-based capacity planning in heterogeneous environments where accuracy at the cell edge directly affects MCS selection and resource block allocation. SVR and polynomial regression are not recommended for throughput prediction: despite SVR’s general nonlinear approximation capability, neither model (*R*^2^ < 0.56) approaches the performance of tree-based or neural approaches on this dataset. The computational overhead of SVR kernel training scales quadratically with the training set size, making it poorly positioned relative to the ANN which scales linearly through stochastic gradient descent. For operators requiring real-time inference on embedded hardware with strict compute budgets, a pruned decision tree may be preferred over the ANN despite the accuracy trade-off, as tree inference requires no floating-point multiply-accumulate operations. ML-based beam management for 5G NR mmWave communications shows similarly strong benefits from neural approaches [42], reinforcing the generality of the ANN-over-shallow-models advantage for nonlinear wireless prediction tasks.

### Physical interpretability

Distance dominance (importance 0.65–0.85) is grounded in the fundamental inverse-square law: a 10% distance error propagates to 18–34% throughput variation, confirming that distance measurement precision should be prioritized in field deployments. In 5G NR, positioning reference signals enable UE location estimates with accuracy of 1–10 m depending on bandwidth and antenna configuration [2]; the sensitivity analysis confirms that 10% distance perturbation produces *R*^2^ degradation below 0.05, suggesting that standard positioning quality is sufficient to maintain model accuracy without dedicated ranging hardware. Frequency importance escalation at mmWave reflects atmospheric physics—oxygen absorption at 60 GHz (≈15 dB/km) and water vapor at 28 GHz (≈1−2 dB/km) [33]—learned implicitly by the ANN through training data without explicit programming. The emergence of this physically motivated frequency dependence from regression on path loss and throughput data, without any explicit absorption term in the feature set, provides a validation of the ANN’s capacity for implicit physics discovery. The low scenario-type importance (< 0.12) is practically useful: it implies that scenario classification can be omitted from lightweight inference pipelines without meaningful accuracy loss. This result suggests that the scenario code functions primarily as a training-time regularizer—ensuring the model is exposed to diverse propagation conditions—rather than an inference-time discriminator that the model actively relies upon.

### Implications of cross-scenario failure on rural environments

The catastrophic RMa failure ($R^{2}<-3$ under urban LOS training) is the most actionable finding of this study. Rural propagation involves terrain-diffraction, low building clutter, and distance-dependent path loss exponents that diverge fundamentally from the urban LOS training distribution. For operators planning heterogeneous networks spanning macro, small-cell, and massive MIMO deployments [43,44], this mandates: (a) mixed-scenario training as the minimum baseline, not an optional enhancement; (b) explicit scenario identification prior to inference to avoid applying a model outside its valid distribution; and (c) dedicated RMa fine-tuning or augmented sampling when rural coverage is a primary objective, as the residual gap ($\Delta R^{2}\approx 0.20$ versus other scenarios) indicates that standard equal-weight mixed sampling underrepresents RMa complexity.

The scenario identification requirement in point (b) deserves elaboration. In practice, scenario boundaries are not hard: a peri-urban deployment may exhibit propagation characteristics intermediate between UMa-NLOS and RMa. A practical safeguard is to include a lightweight LOS/NLOS probability estimator [7] as a preprocessing step, and to flag predictions made with low classifier confidence for human review rather than passing them directly to planning tools. The scalable cell-free massive MIMO architecture considered in [43] and [44], where distributed access points serve users across macro and micro cell boundaries simultaneously, represents a deployment context where the cross-scenario failure identified here is especially dangerous: a single ML channel model serving both urban and rural access points will fail on rural nodes unless explicitly designed for cross-scenario robustness through mixed training and scenario-aware inference gating.

### Practical deployment workflow

The experimental findings translate directly into a recommended workflow for ML-assisted 5G network planning. In the *site selection phase*, linear regression path loss models—calibrated to the log-distance form confirmed by TR 38.901—provide adequate coverage prediction accuracy ($R^{2}\approx 1.0$ on UMi NLOS) at negligible computational cost, enabling rapid evaluation of thousands of candidate sites without prohibitive inference overhead. The 5-feature input (distance, frequency, scenario code, and derived log-transformed features) requires no site-specific measurement data beyond basic geographic classification.

In the *capacity planning phase*, where throughput predictions directly inform resource block allocation and MCS policy, ANN models are required. The 20.15 Mbps RMSE achieved here corresponds to approximately 1% of the peak throughput at short range and 20% at cell edge—within acceptable bounds for planning tools that are subsequently verified by drive tests and field measurements. The ANN should be retrained quarterly using accumulated field measurements to correct for environmental drift, seasonal variation (vegetation effects at sub-6 GHz, rain attenuation at mmWave), and infrastructure changes.

In the *scenario-boundary identification phase*, a lightweight LOS/NLOS classifier [7] should be deployed as a preprocessing step before ANN inference, with predictions flagged for human review when classifier confidence falls below a threshold calibrated to the maximum acceptable cross-scenario *R*^2^ degradation. For deployments where RMa cells coexist with UMa cells within the same planning domain, the residual RMa accuracy gap (*R*^2^ = 0.753 under mixed training) indicates that dedicated RMa fine-tuning is warranted, using a minimum of 500–1,000 field-measured RMa samples to close the $\Delta R^{2}\approx 0.20$ gap relative to urban scenarios.

### Limitations and future directions

**Training data dependency.** All models are trained on 3GPP formula outputs and inherit any systematic biases in those formulas. This means that the ML models primarily learn to reproduce the 3GPP path loss and throughput equations efficiently—they do not discover new channel physics from field measurements. Claims of “deployment realism” in this paper should be understood as “deployment-aligned parameterisation” (via vendor EIRP and antenna specifications) rather than validation against empirical field data. The 3GPP TR 38.901 formulas are themselves extensively calibrated against measurement campaigns [12], so the synthetic labels are not arbitrary, but site-specific anomalies (unusual building geometry, terrain irregularities, vegetation) remain unrepresented. While TR 38.901 is extensively validated, integration of field measurement data via transfer learning or hybrid trained-empirical training is a natural next step.

**Missing advanced baselines.** The present study benchmarks five classical regression architectures. Physics-informed neural networks (PINNs), which encode Maxwell’s equations or log-distance propagation laws as loss constraints, and uncertainty-aware models producing calibrated prediction intervals have demonstrated strong performance in related engineering prediction tasks [26,27] and represent natural extensions that should be compared against the present ANN baseline in future work. Graph-based architectures that represent the spatial network topology explicitly are similarly promising. The present work provides the controlled benchmark dataset and evaluation protocol against which such models should be assessed.

**Geographic diversity.** Current training covers standardized scenario categories but not regional propagation variations within them. Dense megacity and low-rise suburban environments differ substantially in clutter density, building height distribution, and street canyon geometry within the same “Urban Macro” label; the TR 38.901 UMa parametrization represents a statistical average over these conditions rather than any specific urban morphology. Operators deploying in cities with atypical building stock—high-rise clusters, historic dense cores, or waterfront environments with large open-water reflectors—may observe systematic residuals between model predictions and field measurements that cannot be corrected without region-specific training data. Transfer learning from the mixed-scenario pretrained model using a small number of locally collected drive test samples (50–200 measurements) is expected to substantially reduce this regional bias with minimal data collection overhead, and represents a priority direction for operational deployment of the proposed framework.

**Static channel assumption.** The framework models instantaneous channel states and does not account for temporal dynamics, channel non-stationarity, or interference fluctuations—characteristics that become increasingly significant in beyond-5G and 6G scenarios [45]. The static assumption is most limiting in high-mobility scenarios (vehicle speeds > 60 km/h), where the channel coherence time $T_{c}\approx 0.423/f_{D,\max}$ (where $f_{D,\max}$ is the maximum Doppler shift) falls below the subframe duration of 1 ms, invalidating the per-subframe path loss model. Recurrent architectures such as LSTM networks would enable time-series prediction of channel state evolution, but require temporally resolved training datasets pairing consecutive channel measurements with UE trajectories—data not available from the 3GPP TR 38.901 synthetic generation procedure used here.

**Frequency coverage.** The 6G sub-THz bands introduce molecular absorption and near-field effects outside the 3GPP TR 38.901 scope. Physics-informed neural networks incorporating Maxwell’s equations as auxiliary loss constraints may improve extrapolation to these regimes [17].

## Conclusion

This work establishes and validates a comprehensive framework integrating 3GPP TR 38.901 standardized channel models with five machine learning regression architectures for 5G performance prediction. Vendor-calibrated parameterization using Nokia, Huawei, and ZTE equipment specifications ensures alignment with commercial deployment realities, bridging the gap between academic propagation modeling and operational network planning tools.

The principal findings are: (i) for throughput prediction, only ANN-class models achieve satisfactory accuracy (*R*^2^ = 0.998); linear, polynomial, and SVR regressors fail ($R^{2}\leq 0.56$) due to the strongly nonlinear throughput surface arising from the compounding of log-distance path loss, Shannon capacity mapping, and scenario-dependent multipath structure; (ii) for path loss estimation, all models achieve $R^{2}\approx 1.0$, confirming that simple regressors suffice for smooth log-distance targets and that model complexity should be matched to the structural complexity of the target variable rather than applied uniformly; (iii) vendor link budgets quantify the practical performance gap between commercial configurations: Nokia’s 3.5 dBm EIRP advantage nearly doubles throughput over Huawei at 100 m, and ZTE’s 28 GHz deployment delivers 1.6 Gbps at short range with rapid degradation beyond 200 m, validating the densification requirement for FR2; (iv) single-scenario LOS-urban training produces catastrophic failure on Rural Macro ($R^{2}<-3$), mandating mixed-scenario training as the minimum viable baseline for heterogeneous network deployments; and (v) the ANN maintains *R*^2^ > 0.90 under realistic parameter perturbations of ±10% distance, ±5% frequency, and ±2 dB EIRP, confirming deployment suitability without perfect parameter knowledge.

These findings collectively support a tiered modeling strategy for 5G network planning: linear log-distance models for rapid path loss estimation in site selection, ANN models for throughput-based capacity planning in heterogeneous environments, and mixed-scenario training with RMa-augmented sampling for deployments spanning urban and rural cells. The 40–50% RMSE reduction of the ANN over classical Hata and COST-231 models (Table 6) further motivates integration of ML-based prediction tools into standard network planning workflows, where they complement physics-based simulation by enabling fast, trained inference over large parameter spaces.

Future work will integrate field measurement campaigns to supplement 3GPP synthetic training data with site-specific empirical observations, extend the framework to 6G frequency bands above 100 GHz where molecular absorption requires physics-informed neural network architectures, and develop temporal models for dynamic scenarios including vehicle-to-infrastructure and satellite-terrestrial links. The training dataset and model implementations are available upon reasonable request to the corresponding author.

## Supporting information

S1 Table Complete simulation configuration. Carrier frequencies, channel bandwidths, antenna gains, UE receiver sensitivity, noise figure assumptions, and scenario parameters used in dataset generation.
(TIFF)

S2 TableFree-space path loss reference values.Tabulated $PL_{free}$ from Eq 1 for distances 100–1000 m at 1.0, 3.5, and 28.0 GHz.(TEX)

S1 FigFree-space path loss versus distance.Log-scale visualization of $PL_{free}$ at 1, 3.5, and 28 GHz illustrating the 20 dB/decade slope and frequency-dependent offset.(TIFF)
