# Supplementary figures and images for: Machine learning-enhanced 3GPP channel modeling for 5G networks: A vendor-calibrated framework with cross-scenario validation
Source: PLoS One. 2026 Jul 15;21(7):e0353163. doi: 10.1371/journal.pone.0353163 (PMC13372187; doi:10.1371/journal.pone.0353163)

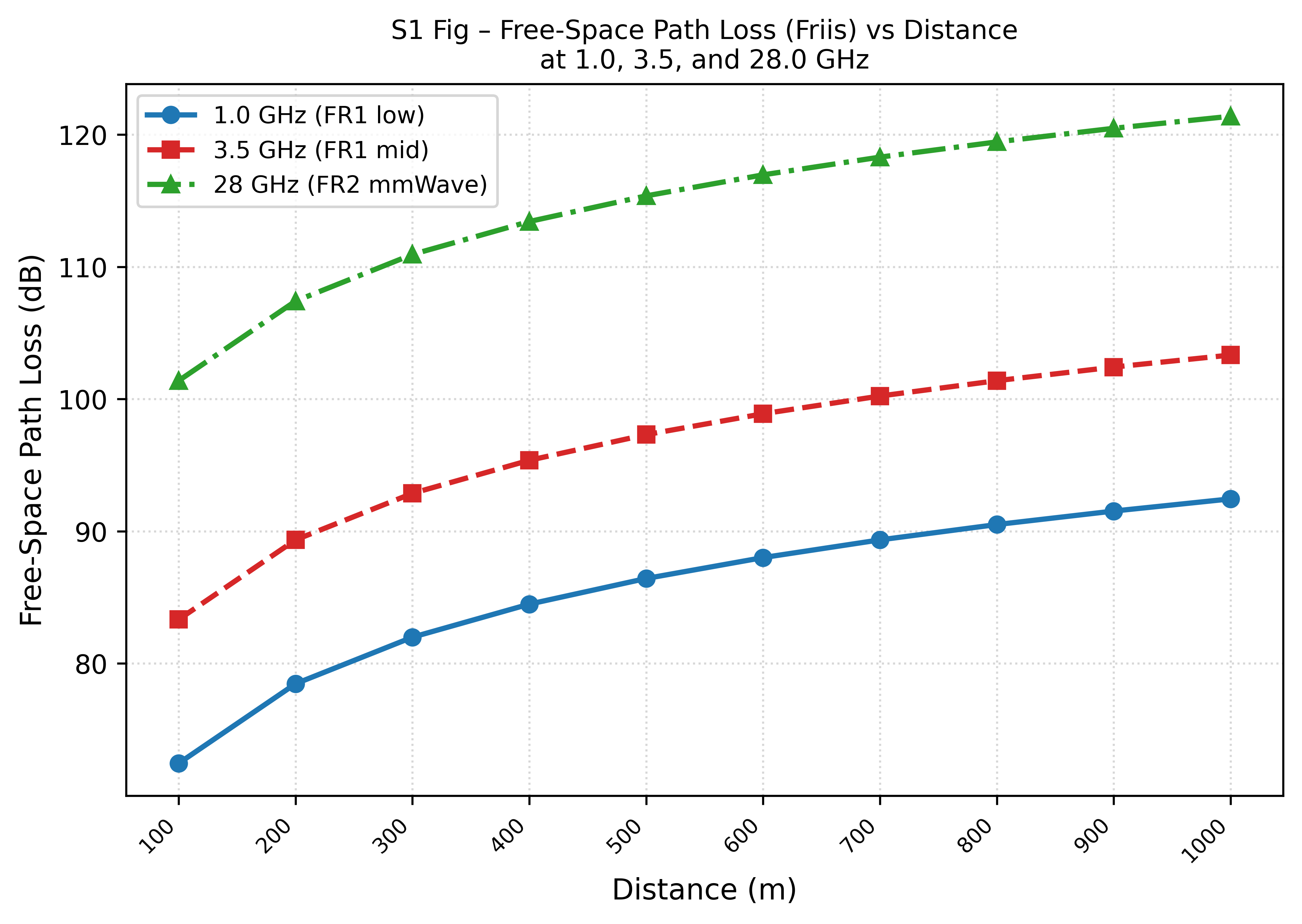

Supplement: S1 Table — Carrier frequencies, channel bandwidths, antenna gains, UE receiver sensitivity, noise figure assumptions, and scenario parameters used in dataset generation. (TIFF) [file pone.0353163.s001.tiff]
