# Supplementary material for: Machine learning-enhanced 3GPP channel modeling for 5G networks: A vendor-calibrated framework with cross-scenario validation
Source: PLoS One. 2026 Jul 15;21(7):e0353163. doi: 10.1371/journal.pone.0353163 (PMC13372187; doi:10.1371/journal.pone.0353163)
Supplement: Attachment — Submitted filename: review report.pdf [file pone.0353163.s004.pdf]

The manuscript combines 3GPP channel models, vendor parameters, and supervised ML for 5G performance prediction. In my view, the present work cannot be accepted in its current form because the ML contribution is too incremental, the validation is largely synthetic, several claims are stronger than the evidence supports. Besides that, there are inconsistencies between tables, text, and figures. The manuscript requires substantial technical strengthening and rigorous re-review before it can be considered further. Some of my detailed comments are as follows,

1. I do not find the ML contribution sufficiently novel. The paper compares standard regressors (linear regression, polynomial regression, SVR, decision tree, and a small MLP) on deterministic 3GPP-generated data. This is closer to supervised curve fitting than a new ML-enhanced channel modeling framework. The authors should clearly state what is new, beyond applying common regressors to simulated data.

2. The entire dataset is generated from 3GPP formulas, yet the manuscript repeatedly claims deployment realism and operational relevance. I find this overstated because no field measurements or independent real-world validation are used. The models mainly learn the equations used to generate the labels.

3. The ML comparison omits stronger state of the art baselines such as physics-informed neural networks, uncertainty-aware models, and graph based deep learning models. For a modern ML paper, this baseline set is not sufficient.

4. On Page 8/Table 2, the ANN is reported with RMSE = 20.15 Mbps and R^2 = 0.998, but Fig. 1 shows the ANN panel with R^2 = 0.753 and RMSE = 247.5. This undermines the confidence in the reported results.

5. The quality of figures needs to be improved. Additionally, Fig. 4 visually contradicts the text. The heatmap values shown are around $R^2 \approx 0.75$, while the manuscript claims $R^2 > 0.90$ under perturbations.

6. Scenario type is treated as an integer code from 0 to 5, which imposes an artificial ordinal relationship between categorical environments. This is not appropriate for ML regression and may distort feature importance and model behavior. Therefore, authors should test one-hot encoding or scenario-specific heads.

7. The paper reports single train/test results but does not provide confidence intervals, statistical significance or learning curves. I would expect at least multiple random splits and uncertainty bands, especially since the paper makes strong claims about model superiority and robustness.

8. While the manuscript provides a broad overview of ML algorithms, it omits several important and recent developments in data-driven approaches for engineering applications. Topics such as physics informed, uncertainty-aware formulations and inverse problem settings are not discussed. Recent contributions in literature (e.g.,

DOI: 10.1016/j.euromechsol.2026.106181 10.1080/15397734.2025.2571730 10.1016/j.tws.2026.114743 10.1007/s41742-026-01049-w 10.1177/07316844251371511 10.1080/15376494.2025.2471036) highlight these emerging directions. I recommend that the authors critically discuss such works to better position their study within the current state of the art.
