# Supplementary material for: Machine learning-enhanced 3GPP channel modeling for 5G networks: A vendor-calibrated framework with cross-scenario validation
Source: PLoS One. 2026 Jul 15;21(7):e0353163. doi: 10.1371/journal.pone.0353163 (PMC13372187; doi:10.1371/journal.pone.0353163)
Supplement: Attachment — Submitted filename: Response_to_Reviewers_auresp_2.docx [file pone.0353163.s007.docx]

**First Response**

**Reviewer 1: Questioning and Answering (In Blue)
Unclear Novelty:** We have added an explicit novelty paragraph in the introduction that distinguishes this work from prior ML-propagation studies: 3GPP anchor data, identical evaluation conditions, and vendor-authenticated parameters.

**Parameter assumption unjustified:** We have added the parameter justification in the method section, explaining why each distance/frequency range and scenario was chosen and mapped to 3GPP TS 38.101 operating envelope.

**Rationale for ML model selection:** Added in the Contribution section: The model spans the full complexity spectrum from closed-form to nonparametric to neural, enabling minimum-complexity selection.

**All the examined models applied in all carried frequencies:** Added explicit confirmation in both abstract and methods dataset sections.

**Quality of the figures:** All the figures are re-rendered in 600 DPI.

**Reviewer 2: Questioning and Answering (In Teal)**

**ML Contribution Novelty:** We reframe the contribution explicitly as “benchmarking, structure, and vendor calibration, not new channel model discovery.”

Deployment realism overstated: We replace “ensure deployment realism” with “deployment-aligned (formula-derived, not field-measured)” throughout the abstract, introduction, and limitations.

**Missing SOTA baseline**: Added a full paragraph in Related Work covering PINNs, uncertainty-aware models, and graph-based DL with proper citations; added a "Missing advanced baselines" subsection in Limitations.

**Figure 1 vs. Table 2 inconsistency:** Added an explanatory paragraph clarifying Fig. 1 shows full mixed-scenario distribution (including out-of-distribution RMa samples), while Table 2 shows in-distribution split; both are correct and consistent.

**Figure 4 heatmap Correction:** Revised Fig 4 caption to clarify heatmap shows absolute R² per scenario subset; R²≈0.75 cells are RMa subsets (consistent with Table 4); urban/indoor subsets remain >0.90.

**Cross-validation:** Added 5-fold random-split protocols to the method and also Table 2 now reports mean ± 95% CI for all metrics.

**Missing recent literature:** All 6 DOIs added and cited in Related Work and Limitations.

In addition to all of that, all the datasets used in this article are now publicly available at <https://zenodo.org/records/20051347,> and to meet PLOS ONE’s style requirement, we use the journal template from <https://journals.plos.org/plosone/s/latex>.

**Second Response:**

1 = Ok

2 = Ok

3 = Ok

4 = Ok

6= Reviewer 1: Ok.

Reviewer 2: All the DOI issues have been solved.
